# Supplementary material for: Enhanced Magnetic Behavior of Cobalt Nano-Rods Elaborated by the Polyol Process Assisted with an External Magnetic Field
Source: Nanomaterials (Basel). 2020 Feb 15;10(2):334. doi: 10.3390/nano10020334 (PMC7075111; doi:10.3390/nano10020334)
Supplement: Supplementary file 1 [file nanomaterials-10-00334-s001.pdf]

nanomaterials

Supplementary Material

Enhanced Magnetic Behavior of Cobalt Nano-Rods Elaborated by the Polyol Process Assisted with an External Magnetic Field

Mohamed Ali Bousnina [1], Amel Dakhlaoui-Omrani [1,2], Frédéric Schoenstein [1], Yaghoub Soumare [1,3], Aliou Hamady Barry [1,3], Jean-Yves Piquemal [4], Guillaume Viau [5], Silvana Mercone [1,*] and Noureddine Jouini [1,*]

[1] Laboratoire des Sciences des Procédés et des Matériaux, CNRS, UPR 3407, Université Sorbonne Paris Nord, 99 avenue J.B. Clément, F-93430 Villetaneuse, France; medalibousnina@yahoo.fr (M.A.B.); amel_dakhlaoui@yahoo.fr (A.D.-O.); frederic.schoenstein@univ-paris13.fr (F.S.); soumareyaghoub@yahoo.fr (Y.S.); barryaliouhamady@ymail.com (A.H.B.)
[2] Department of Chemistry, Faculty of Sciences and Arts-Khulais, University of Jeddah, Khulais P.O. Box 355, Jeddah 21921, Saudi Arabia
[3] Laboratoire de chimie des Matériaux, Département de chimie, Faculté des sciences et Techniques, Université des Sciences de Technologie et de Médecine, Nouakchott BP 880, Mauritanie
[4] Laboratoire Interfaces Traitements Organisation et DYnamique des Systèmes, CNRS, UMR 7086, Université de Paris, 15 rue J.-A. de Baïf, F-75013 Paris, France; jean-yves.piquemal@u-paris.fr
[5] Laboratoire de Physique et Chimie des Nano-objets, CNRS, INSA, UPS, Université de Toulouse, 135 avenue de Rangueil, F-31077, Toulouse CEDEX 4, France; guillaume.viau@insa-toulouse.fr
* Correspondence: silvana.mercone@univ-paris13.fr (S.M.); jouini@univ-paris13.fr (N.J.); Tel.: +33-1-49-40-34-20 (S.M.); +33-1-49-40-34-35 (N.J.)

Received: 31 December 2019; Accepted: 11 February 2020; Published: 15 February 2020

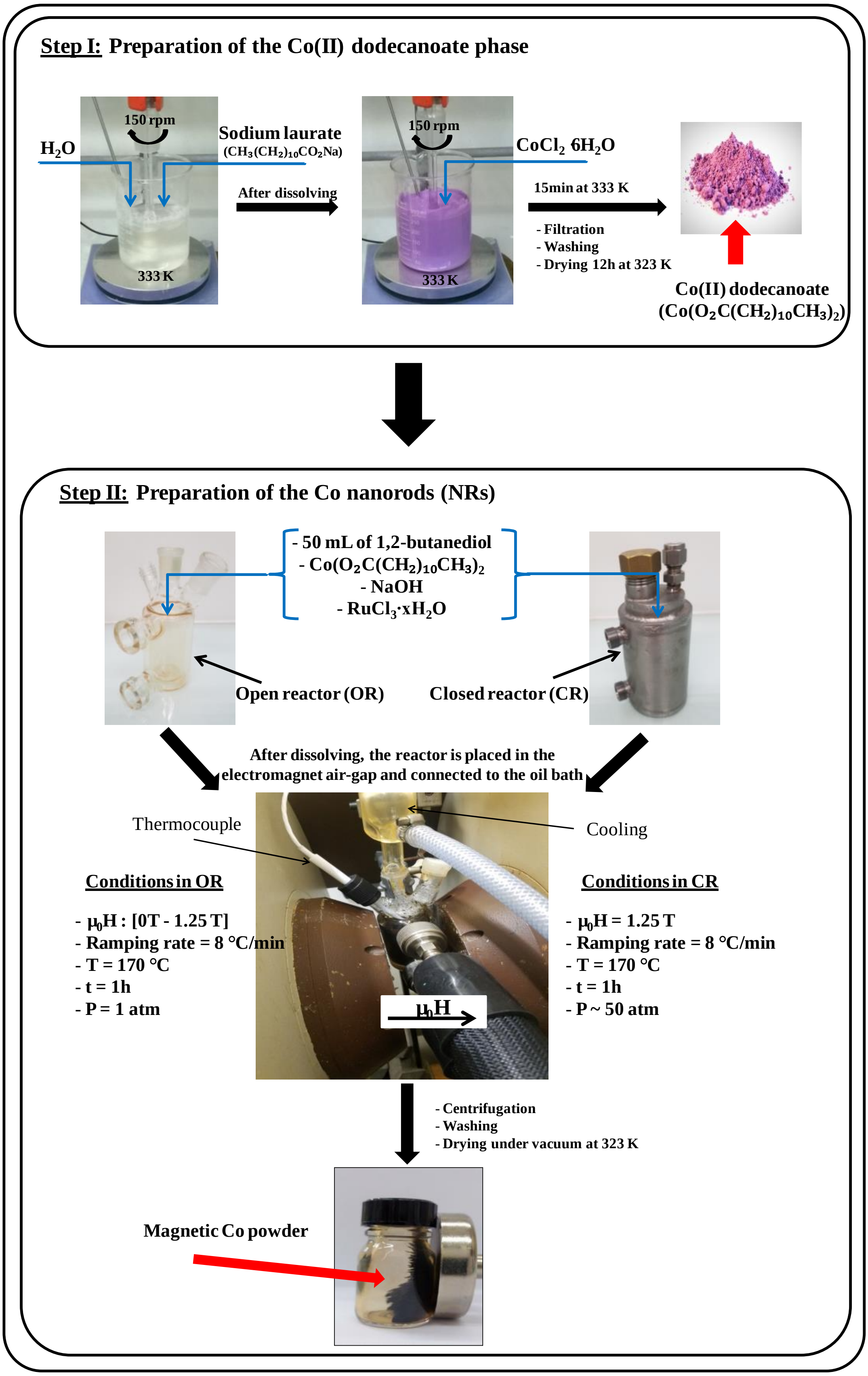

Figure S1. Schematic drawing of the setup and of the preparation steps of Co NWs in open reactor / closed reactor under external magnetic field.

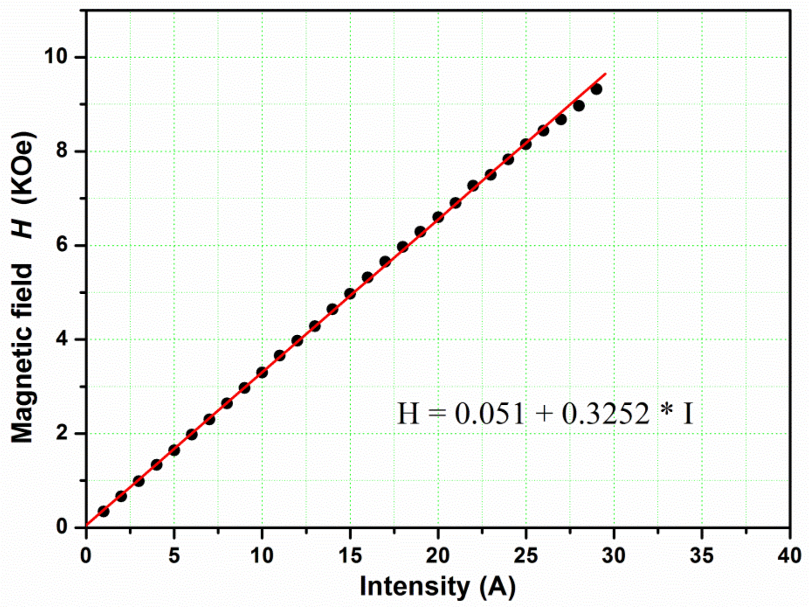

Figure S2. Calibration curve of the electromagnet: Magnetic Field versus Intensity.

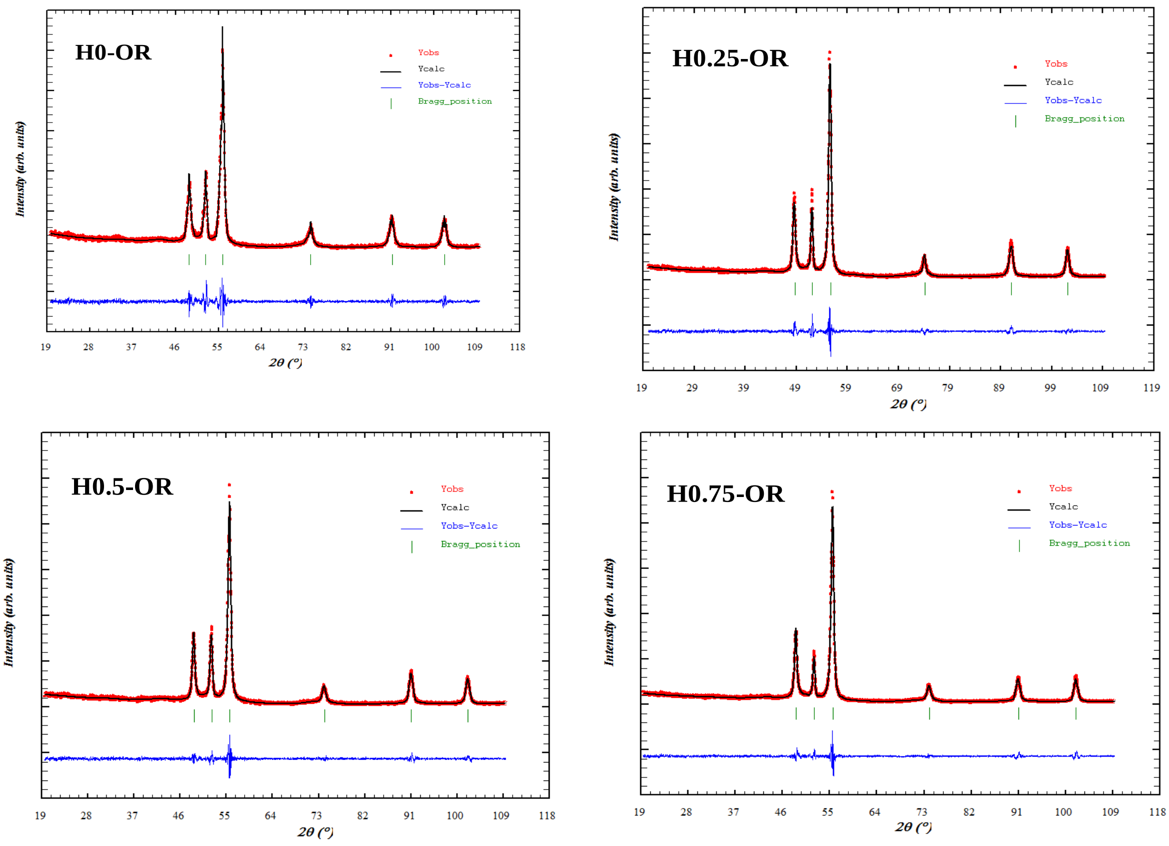

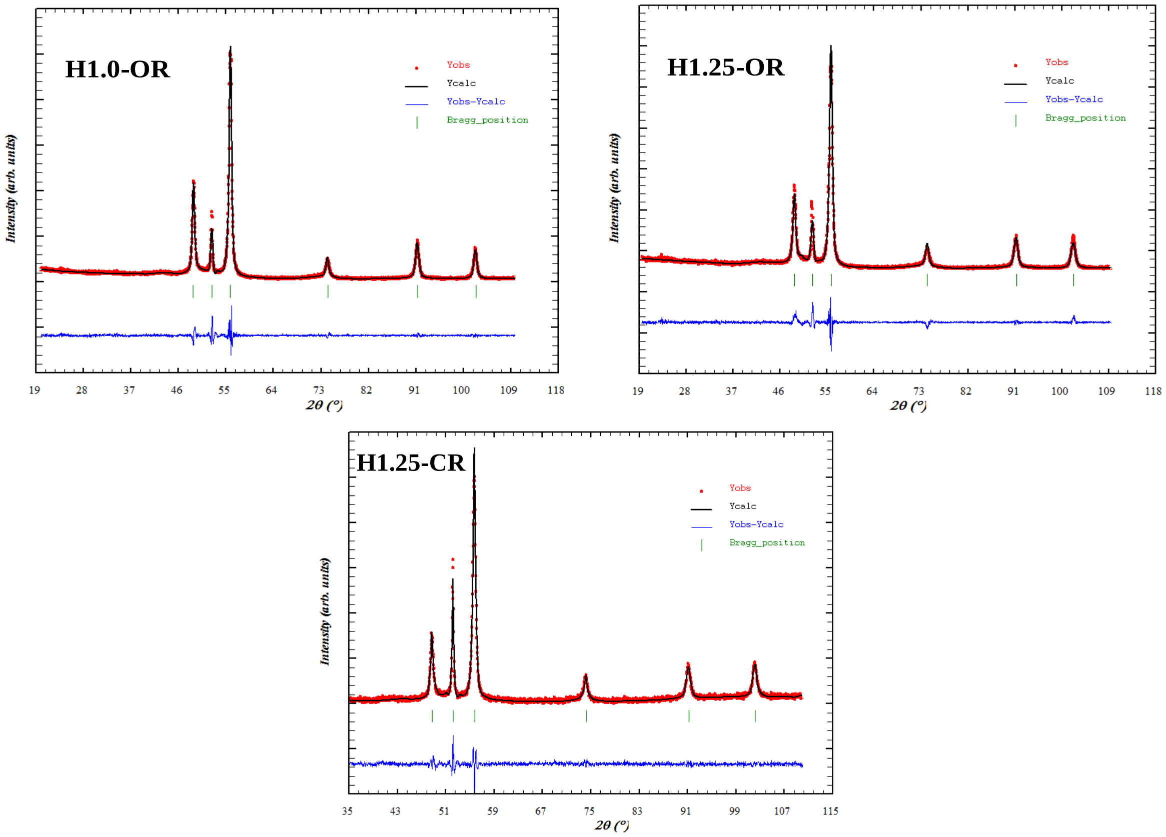

Figure S3. Rietveld refinement results of XRD patterns of Co NWs samples prepared under external magnetic field varied from 0 T to 1.25 T. The experimental data are shown as dots; the global fitting profile and the difference curve are shown as solid lines; the calculated reflection positions are indicated by stick marks.

© 2020 by the authors. Submitted for possible open access publication under the terms and conditions of the Creative Commons Attribution (CC BY) license (http://creativecommons.org/licenses/by/4.0/).
